# General and Vulnerable Population’s Satisfaction With the Healthcare System in Urban and Rural Areas: Findings From the European Social Survey

**DOI:** 10.3389/ijph.2022.1604300

**Published:** 2022-03-08

**Authors:** Lorenzo Righi, Stéphane Cullati, Pierre Chopard, Delphine S. Courvoisier

**Affiliations:** ^1^ Quality of Care and Clinical Networks, Health Directorate of the Tuscany Region, Florence, Italy; ^2^ Quality of Care Unit, University Hospitals of Geneva, Geneva, Switzerland; ^3^ Department of General Internal Medicine, Rehabilitation and Geriatrics, University of Geneva, Geneva, Switzerland; ^4^ Population Health Laboratory (#PopHealthLab), Department of Community Health, Faculty of Science and Medicine, University of Fribourg, Fribourg, Switzerland

**Keywords:** healthcare system, vulnerability, satisfaction, urban, rural, Europe

## Abstract

**Introduction:** Access to the healthcare system when patients are vulnerable and living outside metropolitan areas can be challenging. Our objective was to explore healthcare system satisfaction of urban and rural inhabitants depending on financial and health vulnerabilities.

**Methods:** Repeated cross-sectional data from 353,523 European citizens (2002–2016). Multivariable associations between rural areas, vulnerability factors and satisfaction with the healthcare system were assessed with linear mixed regressions and adjusted with sociodemographic and control factors.

**Results:** In unadjusted analysis, the people who lived in houses in the countryside and those who lived in the suburbs were the most satisfied with the healthcare system. In the adjusted model, residents living in big cities had the highest satisfaction. Financial and health vulnerabilities were associated with less satisfaction with the healthcare system, with a different effect according to the area of residence: the presence of health vulnerability was more negatively correlated with the healthcare system satisfaction of big city inhabitants, whereas financial vulnerability was more negatively correlated with the satisfaction of those living in countryside homes.

**Conclusion:** Vulnerable residents, depending on their area of residence, may require special attention to increase their satisfaction with the healthcare system.

## Introduction

The purpose of a healthcare system is to cure people and improve their physical and mental well-being, thus ensuring the best attainable average level of health and the smallest possible differences in quality of care between individuals and groups [1]. Satisfaction with healthcare received is a key element of a patient-centred healthcare system [2, 3]. Satisfaction with the healthcare system should be high regardless of place of residence of the patients, both for the general population and for its most vulnerable members. Moreover, in ageing societies [4], the general population is living longer [5], including longer in good general health [6], but a non-negligible part of the population will experience morbidities or multimorbidity [7, 8] and thus will be repeatedly in contact with the healthcare system.

In the general population, satisfaction with the healthcare system is generally moderate in western countries [9, 10] and is related to individual factors on the one hand and to macro (country-level and health system-level) factors on the other, with significant variations between countries [9–11].

Being a woman [9, 10, 12], not having a comfortable income [10, 12–14], and having a poor self-reported health [9–13, 15] or a sadness-related personality trait [11] are individual factors associated with lower satisfaction with the healthcare system. Levels of satisfaction by age depicts a U-shaped curve, with the lowest satisfaction observed among middle-aged individuals [9, 10, 12]. Previous positive experiences of healthcare are associated with higher satisfaction with the healthcare system [11, 13]. However, inconsistent associations have been observed for education, with lower [9, 11, 12, 16] and higher [10] educational achievement being associated with higher satisfaction.

At the macro level, factors linked with lower level of satisfaction are a low number of general practitioners per 1,000 inhabitants [10, 13], high medical cost per family [15], physical distance from health facilities [17] and, more generally, difficult access to care [15, 18, 19]. Inconsistent findings have been observed with the total level of health expenditure in the country [10, 13]. Satisfaction level is negatively affected by the presence of socioeconomic or health vulnerability. Citizens with incomes below the national median are more likely than those with higher incomes to be dissatisfied [20, 21] and to experience health disparities [21–23]. Dissatisfaction [24–26] and health disparities [23, 27, 28] have also been documented for many long-term health conditions. Instead, we do not know whether perception of healthcare quality [29, 30] and healthcare system satisfaction [14, 16, 31–34] are higher for people living in urban or rural areas. What is known is that he density of healthcare facilities and health personnel is higher in urban and wealthier areas [22, 35–39], that even in countries where most of the population lives in rural areas, the healthcare resources are concentrated in the cities [40] and that rural residents in Europe have more problems with access to care than do urban residents [31, 32, 41–43].

To the best of our knowledge, differences in satisfaction of the vulnerable inhabitants of rural and urban areas are not known. The objective of the present study was to examine whether financial and health vulnerabilities moderate the association between living in urban and rural areas and healthcare system satisfaction in Europe.

## Methods

This study used data from the European Social Survey (ESS), a cross-sectional population-based survey repeated every 2 years since 2002, whose objective is to monitor social change in Europe. Respondents were selected using multi-stage random probability sampling to be nationally representative of the residents aged 15 and older and living in households.

The present study considered eight waves, from 2002 to 2016 [44], involving 32 European countries: Austria, Belgium, Bulgaria, Croatia, Cyprus, Czech Republic, Denmark, Estonia, Finland, France, Germany, Greece, Holland, Hungary, Iceland, Ireland, Israel, Italy, Lithuania, Luxembourg, Norway, Poland, Portugal, Russia, Sweden, Slovenia, Slovakia, Spain, Switzerland, Turkey, Ukraine, United Kingdom. Response rates ranged from about 30 to 75% across ESS waves and countries [45, 46]. Most countries did not participate in all the rounds. The sample consisted of 374,729 residents. Respondents with missing data on the outcome (healthcare system satisfaction) (N = 5,090; 1.4% of the initial sample), area of residence (N = 1,103; 0.3%), financial and health vulnerability variables (N = 7,911 + 1,928; 2.1 and 0.5%), and control variables (age N = 1,682, sex N = 332, life satisfaction N = 2,038, education N = 4,090) were excluded from the analysis (total N = 21,206; 5.7% of the initial sample). The percentage included in the final sample ranged from 94 to 96% of the initial sample for the five categories of the area of residence. The final sample used for analysis included 353,523 individuals.

### Dependent Variable

Healthcare system satisfaction was evaluated with the question: “Please say what you think overall about the state of health services in (country) nowadays?”; answers ranged from 0 “Extremely bad” to 10 “Extremely good.”

### Main Independent Variables

Respondents’ area was defined on the basis of the following question: “Which phrase (on this card) best describes the area where you live?”. Five answers were possible: 1) a big city, 2) the suburbs or outskirts of a big city, 3) a town or a small city, 4) a country village, 5) a farm or home in the countryside. We defined vulnerability as a lack of resources [47] and reserves [48] whereby individuals or groups are unable to cope effectively with stressors, be they economic or physiological [48, 49]. In this analysis, two vulnerability variables were used: 1) financial vulnerability, based on the question: “Which of the descriptions on this card comes closest to how you feel about your household’s income nowadays? Living comfortably on present income; coping on present income; finding it difficult on present income; finding it very difficult on present income”; 2) health vulnerability, based on the question: “Are you hampered in your daily activities in any way by any longstanding illness, or disability, infirmity or mental health problem?” with yes or no answer categories. If yes, respondents additionally answered the following question: “is that a lot or to some extent?” with answer categories “Yes, a lot,” “Yes, to some extent” and “No.”

### Control Variables

Control variables were age, sex, education, life satisfaction, country, and year of survey. Age was assessed using five categories: 15–20, 21–35, 36–49, 50–64, and ≥65 years [9]. Education was measured as the number of years in education. Life satisfaction was based on the following question: “All things considered, how satisfied are you with your life as a whole nowadays? Please answer using this card, where 0 means extremely dissatisfied and 10 means extremely satisfied.” In the analyses, education and life satisfaction were treated as continuous variables. Survey round was a variable ranging from 1 (2002) to 8 (2016).

### Statistical Methods

Participants’ characteristics were described using descriptive statistics [mean, standard deviation (SD), frequency and percentages]. They were compared between areas using ANOVA or chi-square test. The association of area of residence with healthcare system satisfaction was analysed by using multivariable linear mixed models with a random intercept for the country. To test whether the association between area of residence and healthcare system satisfaction was moderated by a financial or a health vulnerability factor, the same multivariable linear mixed model was used but including interaction terms. The marginal mean of healthcare system satisfaction derived from this adjusted model was used to create interaction plots of areas of residence and vulnerabilities. Analyses were adjusted for control variables. Models were estimated by using weights provided by ESS (anweight), which corrects for differential selection probabilities as specified by country sample design, non-response, non-coverage, sampling error (based on four post-stratification variables: sex, age, education and geographical region), and variation of population size across countries [50].

All analyses involved using R 4.0.2 (https://www.r-project.org).

## Results

Towns or small cities and country villages were where most respondents lived (31 and 30%); the third was a big city (22%) followed by the suburbs or outskirts of a big city (11.5%) and finally by a farm or home in the countryside (6%).

Socio-demographic characteristics of participants living in the five different areas were quite similar (Table 1). The inhabitants of the big cities were slightly younger and more likely women than the others and they studied as much as the residents in the outskirts of a big city and more than the other groups. They were as satisfied with life as were residents in small cities and country villages but less than people living in the suburbs or outskirts of a big city and in a farm or home in the countryside. Because of the very large sample size, all *p*-values for the tests comparing resident characteristics across domicile were highly significant (all *p*s < 0.001).

**TABLE 1:** Characteristics of respondents according to their area of residence, European Social Survey, 32 European countries, 2002–2016.

	All respondents	Area of residence				
		A big city	The suburbs or outskirts of a big city	A town or a small city	A country village	A farm or home in the countryside
Number of participants	353,523	77,865	40,536	107,993	106,070	21,059
	N (%)	N (%)	N (%)	N (%)	N (%)	N (%)
Women	190,271 (53.8)	43,262 (55.6)	21,369 (52.7)	58,791 (54.4)	56,457 (53.2)	10,392 (49.3)
Age (years)						
15 to 20	23,787 (6.7)	4,991 (6.4)	2,604 (6.4)	7,645 (7.1)	7,315 (6.9)	1,232 (5.9)
21 to 35	78,738 (22.3)	21,255 (27.3)	8,997 (22.2)	24,132 (22.3)	21,127 (19.9)	3,227 (15.3)
36 to 49	85,296 (24.1)	17,948 (23.1)	10,123 (25.0)	26,039 (24.1)	25,937 (24.5)	5,249 (24.9)
50 to 64	88,373 (25.0)	17,735 (22.8)	10,051 (24.8)	26,973 (25.0)	27,449 (25.9)	6,165 (29.3)
≥65	77,329 (21.9)	15,936 (20.5)	8,761 (21.6)	23,204 (21.5)	24,242 (22.9)	5,186 (24.6)
Life satisfaction, mean (SD)	6.9 (2.3)	6.6 (2.4)	7.1 (2.2)	6.8 (2.3)	6.9 (2.3)	7.6 (2.0)
Number of years of education, mean (SD)	12.3 (4.1)	13.0 (4.2)	13.0 (4.1)	12.4 (3.9)	11.5 (4.0)	12.0 (3.9)
Financial vulnerability						
Living comfortably on present income	99,649 (28.2)	17,913 (23.0)	15,145 (37.4)	29,860 (27.6)	29,021 (27.4)	7,710 (36.6)
Coping on present income	158,153 (44.7)	35,002 (45.0)	17,229 (42.5)	48,668 (45.1)	47,261 (44.6)	9,993 (47.5)
Finding it difficult on present income	67,930 (19.2)	17,599 (22.6)	5,970 (14.7)	21,134 (19.6)	20,644 (19.5)	2,583 (12.3)
Finding it very difficult on present income	27,791 (7.9)	7,351 (9.4)	2,192 (5.4)	8,331 (7.7)	9144 (8.6)	773 (3.7)
Health vulnerability						
Not hampered in daily activities	262,719 (74.3)	59,300 (76.2)	30,709 (75.8)	79,643 (73.7)	77,672 (73.2)	15,395 (73.1)
Hampered in daily activities by longstanding illness	68,680 (19.4)	14,022 (18.0)	7,513 (18.5)	21,275 (19.7)	21,585 (20.3)	4,285 (20.3)
A lot hampered in daily activities by longstanding illness	22,124 (6.3)	4,543 (5.8)	2,314 (5.7)	7,075 (6.6)	6813 (6.4)	1379 (6.5)
Outcome						
Healthcare system satisfaction, mean (SD)	5.3 (2.6)	5.1 (2.6)	5.4 (2.5)	5.2 (2.6)	5.3 (2.6)	5.6 (2.5)

For vulnerabilities, the highest proportion of people finding it very difficult to live with their income was in the big cities and the lowest in the group living in farms or homes in the countryside. People living in small cities, in country villages and in the countryside were more frequently hampered in daily activities (proportions ranging from 6.4 to 6.6%), whereas the outskirts group of inhabitants was the least frequently hampered (5.7%).

For area of residence, the inhabitants of big cities and small cities were less satisfied on average than were people living in other areas.

### Factors Associated With Healthcare System Satisfaction

Table 2 presents the multivariable estimates for all variables. Supplementary Table S1 presents the standardised estimates. Women had a more negative perception of the healthcare system than did men. Citizens who were young (15–35 years old) or old (≥65 years old) had a more positive opinion of the healthcare system than did those 36–49 years old (reference category). The coefficient for the number of years of education was slightly negative, which indicates that for each year of study, mean satisfaction decreased, but the difference was very small. Life satisfaction was positively correlated with healthcare system satisfaction and presented the largest standardized estimate.

**TABLE 2:** Multivariable linear mixed regression of area of residence and vulnerability factors associated with satisfaction with the healthcare system, European Social Survey, 32 European countries, 2002–2016.

	Estimate (Std Error)	*p*-Value
(Intercept)	3.78 (0.20)	<0.0001
Female (reference: male)	−0.21 (0.01)	<0.0001
Age (years) (reference: 36–49)		
15 to 20	0.66 (0.01)	<0.0001
21 to 35	0.16 (0.01)	<0.0001
50 to 64	−0.06 (0.01)	<0.0001
≥65	0.24 (0.01)	<0.0001
Years of education	−0.03 (0.00)	<0.0001
Life satisfaction	0.22 (0.00)	<0.0001
Time (survey round)	0.05 (0.03)	0.068
Domicile (reference: big city)		
The suburbs or outskirts of a big city	−0.11 (0.01)	<0.0001
A town or a small city	−0.07 (0.01)	<0.0001
A country village	−0.04 (0.01)	<0.0001
A farm or home in the countryside	−0.14 (0.02)	<0.0001
Financial vulnerability		
“Coping on present income”	−0.17 (0.01)	<0.0001
Compared with “Living comfortably on present income”		
“Finding it difficult on present income”	−0.05 (0.01)	<0.0001
Compared with “Coping on present income”		
“Finding it very difficult on present income”	−0.19 (0.02)	<0.0001
Compared with “Finding it difficult on present income”		
Health vulnerability		
" Hampered in daily activities”	−0.12 (0.01)	<0.0001
Compared with “Not hampered in daily activities”		
“A lot hampered in daily activities”	−0.02 (0.02)	0.26
Compared with “Hampered in daily activities”		

Note: The above model was estimated using sampling weights and adjusted by country.

For area of residence, in a multivariable model controlling for sociodemographic and control factors, inhabitants of big cities were more satisfied than were people living in other areas.

Financial and health vulnerabilities were both significantly and inversely associated with healthcare system satisfaction, with financial vulnerability having a stronger negative impact.

We found no clear evidence of the association of the time variable (survey rounds, 2002–2016) in a model adjusted for sociodemographic and control factors.

### Moderation of Vulnerability Factors

**FIGURE 1:**
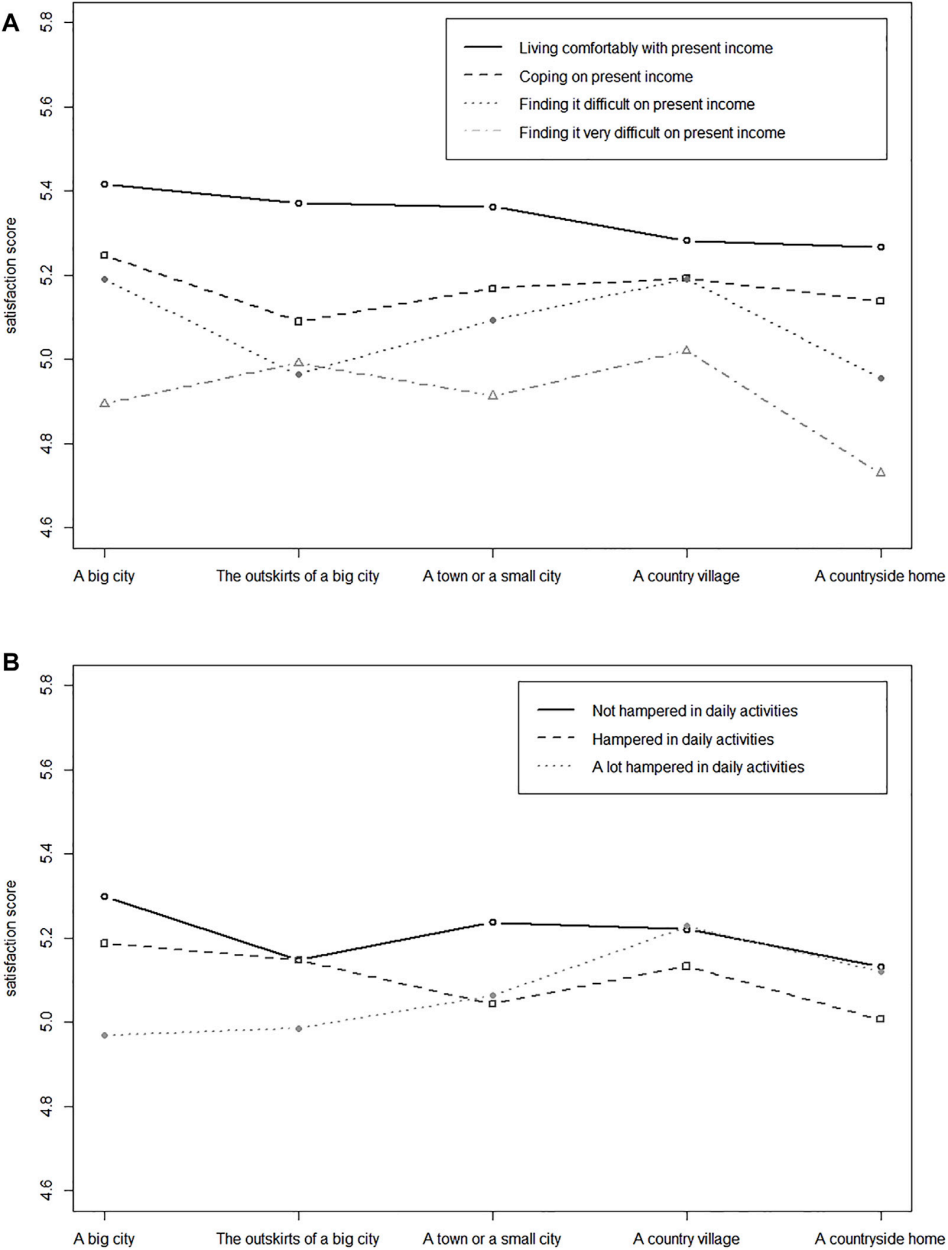
**(A)** Interaction plot of areas of residence and financial vulnerability on satisfaction with the healthcare system, European Social Survey, 32 European countries, 2002–2016. **(B)** Interaction plot of areas of residence and hampering condition vulnerability on satisfaction with the healthcare system, European Social Survey, 32 European countries, 2002–2016.

Figure 1 shows healthcare system satisfaction of residents in different types of areas and in the presence of vulnerabilities. The interaction between area of residence and the presence of a financial vulnerability as well as the interaction between area of residence and the presence of a hampering condition vulnerability on satisfaction were both highly significant (*p*s < 0.001).

The figure allows for two comparisons: the satisfaction of vulnerable people in the different areas of residence (lower lines) and the satisfaction gap (the distance between the top and the bottom lines) in the five areas of residence.

People with financial vulnerability (Figure 1A) who were living in a countryside home were less satisfied than were people with financial vulnerability living in other areas. Country villagers were the most satisfied. In all areas, we found a gradient of satisfaction related to the degree of economic difficulty. The presence of financial vulnerability resulted in a small healthcare system satisfaction gap among country villagers, a larger gap among the inhabitants of the outskirts of a big city and small cities, and an even larger gap among people living in big cities and the countryside.

People hampered greatly in daily activities (Figure 1B) who were living in a big city or the outskirts of a big city were less satisfied than were people with a hampering condition vulnerability living in other areas. Country villagers were the most satisfied. We found no clear gradient of satisfaction in areas of domicile, with the exception of residents of big cities. In all areas, the satisfaction gap was smaller than that generated by the presence of a financial vulnerability. Big cities had the largest gap.

## Discussion

This repeated cross-sectional study analysed the healthcare system satisfaction of 353,523 individuals from 32 European countries and showed the association of areas of residence and vulnerability factors with healthcare system satisfaction.

### Main Findings

At the descriptive level, the areas of residence in descending order of satisfaction were “a farm or home in the countryside,” “the suburbs or outskirts of a big city,” “a country village,” “a town or small city,” and “a big city.” The proportion of people finding it very difficult to live on their present income was highest in big cities and lowest in homes in the countryside. The proportion of people hampered a lot in daily activities by illness was highest in small cities and lowest in the outskirts of big cities.

In the model adjusted for sociodemographic variables (sex, age, education), life satisfaction and vulnerability factors, we found a pattern opposite to the descriptive analysis, with the inhabitants of big cities the most satisfied. People living in country villages and small cities had an intermediate satisfaction. People living in the outskirts of a big city or in a home in the countryside had the lowest satisfaction. Middle-aged people (36–49 years), females and those with higher number of years of study had a lower level of healthcare system satisfaction, whereas higher life satisfaction was positively associated with healthcare system satisfaction, as previously shown in the literature [9, 10, 12, 13]. We acknowledge that differences between areas of residence were small in the scale level of the outcome, as illustrated by the graphs.

The two vulnerability variables were negatively associated with healthcare system satisfaction, with the presence of financial problems having a stronger negative impact than a hampering condition. The impact of vulnerabilities on healthcare system satisfaction varied according to areas of residence (Figure 1). Country house dwellers and inhabitants of big cities with financial vulnerability exhibited the largest gap in healthcare system satisfaction, but the effect was more moderate for people living in the outskirts of a big city and in country villages. The most negative impact with a hampering condition vulnerability was found in big cities and the least negative in rural areas.

### Limitations

First, our large sample presents statistically significant effects for almost all variables under study, but these effects are not necessarily meaningful. Second, the outcome was the overall satisfaction with the healthcare system. This measure is an umbrella indicator implying different facets, identified in previous studies [51]. Third, we were unable to adjust our model with known predictors of healthcare system satisfaction, like previous patient experiences with health care [11]. Fourth, we cannot exclude an effect of people’s inherent rating tendency affecting the reported healthcare system satisfaction score; however, as for patient satisfaction scores, this adjustment could be marginal [52]. Fifth, we did not analyse how urban areas differed between European countries: large unstudied variations are possible and depending on the country of residence may change the interpretation of the area (urban or rural) where the person lives. Sixth, we were unable to know how long survey participants had lived in their area of residence. Seventh, differences in satisfaction could be driven by people’s interpretation of the question varying by urbanity, although differences in interpretation should be mitigated by the models used in the article being adjusted for sex, age, education, and country.

### Interpretation

Our findings lead to several considerations. First, using five types of residence areas, this study has an accurate view of healthcare system satisfaction, highlighting differences that would otherwise not be detectable. Big cities are different from small ones and suburbs are different from city centres, just as country villages are different from farms or homes in the countryside. Living in these areas seems to affect the level of satisfaction with the healthcare system and, notably, is independent of the (sociodemographic, socioeconomic and life satisfaction) characteristics of individuals.

The distribution of the factors influencing healthcare system satisfaction is heterogenous (Table 1); for this reason the inhabitants of the big cities were the least satisfied in the descriptive analysis (Table 1) and the most satisfied in the statistical model (Table 2). In our model, the variable with the greatest influence on healthcare system satisfaction was life satisfaction.

The presence of vulnerabilities had a negative impact on healthcare system satisfaction. This finding may not be surprising [20, 21, 24–26], but the most fragile people being the least satisfied with their healthcare system indicates that European health systems are not completely fulfilling their mission. The biggest gap between non-vulnerable and vulnerable inhabitants’ satisfaction was found in big cities. The smallest gap was found in rural villages, where people with financial vulnerability were slightly less satisfied than those without financial vulnerability and where those with a hampering condition were as satisfied as the rest of the population. Satisfaction of the country villagers deserves further studies, considering that in our model (Table 2), the healthcare system satisfaction of the general population living in country villages is second only to that of those living in large cities.

Several explanations for healthcare system satisfaction are possible. Satisfaction can be related to the quality of primary care, which may be equal or higher in rural than urban areas [29, 34, 53]. Or, transferring what Lenzi and Perucca studied [54] into the healthcare field, proximity to large cities and therefore accessibility to their agglomeration advantages may help in understanding the healthcare system satisfaction of residents in smaller cities. These two elements may coexist, and therefore the basic needs of citizens may be well handled by primary care and local hospitals, whereas the more complex needs are handled by tertiary hospitals that may not be nearby but within relatively easy reach [55–57].

Further studies are needed to understand the difference in satisfaction between country villages and country homes, the latter being the area with the lowest level of satisfaction in our multivariable model (Table 2), regardless of the other factors considered. The difference in level of satisfaction may be related to inadequate quality, quantity or distribution of primary care providers [19, 43, 58–60]; to a greater difficulty in accessing local or tertiary hospitals [61, 62]; or to a voluntary reduced use of health care services [42, 60, 63]. Rural villagers’ satisfaction may also be related to the supportive role of small communities [60, 61] that may be absent in people who live more isolated. Of note, even for people living in country homes, a financial vulnerability has a more negative impact than a health vulnerability (Figures 1A,B). To use the healthcare system satisfactorily, countryside home residents must have good economic resources and to a greater extent than country village residents.

A further critical issue is that the satisfaction of European citizens did not improve from 2002 to 2016. This finding shows that there is still work to be done regarding healthcare system satisfaction, despite the focus on the issue in recent years.

A final consideration: European health systems respond more effectively to the needs of non-vulnerable citizens in the centre of big cities. In doing so, they not only do not respond to the needs of the most fragile minorities (citizens with vulnerabilities) but they also do not even respond to the needs of the majority because most European citizens do not live in the centre of big cities. Instead, they respond to the needs of a privileged minority who do not believe that the healthcare system is adequate for their needs.

Our study shows that healthcare system satisfaction in Europe varies by domicile and that the presence of financial or health vulnerabilities has a different impact in relation to where the citizen lives. People living in the suburbs of a big city or in a home in the countryside and vulnerable people living in big cities are the least satisfied. These findings raise concerns about inequality in European healthcare systems and indicate the need to rethink healthcare systems to guarantee that everyone the same access to care and quality of care regardless of the place of residence and conditions of fragility.

## Data Availability

Data from the ESS is publicly available (https://www.europeansocialsurvey.org/), which is how we got access to it. ESS data are licenced under the creative commons “Attribution-NonCommercia-ShareAlike 4.0 International” (CC BY-NC-SA 4.0). Access to the data requires a registration.
