# Supplementary material for: General and Vulnerable Population’s Satisfaction With the Healthcare System in Urban and Rural Areas: Findings From the European Social Survey
Source: Int J Public Health. 2022 Mar 8;67:1604300. doi: 10.3389/ijph.2022.1604300 (PMC8938939; doi:10.3389/ijph.2022.1604300)
Supplement: Supplementary file 1 [file Table1.docx]

**Table S1 – Standardised multivariable linear mixed regression of area of residence and vulnerability factors associated with satisfaction with the healthcare system, European Social Survey, 32 European countries, 2002-2016**

|  | Standardised Estimate  (Std Error) | p-value |
| --- | --- | --- |
| Female | -0.04 (0.00) | <0.0001 |
| *Age (years)* |  |  |
| 15 to 20 | 0.06 (0.00) | <0.0001 |
| 21 to 35 | 0.03 (0.00) | <0.0001 |
| 50 to 64 | -0.01 (0.00) | <0.0001 |
| ≥65 | 0.04 (0.00) | <0.0001 |
| Years of education | -0.05 (0.00) | <0.0001 |
| Life satisfaction | 0.20 (0.00) | <0.0001 |
| Time (survey round) | 0.04 (0.02) | 0.07 |
| *Domicile* |  |  |
| The suburbs or outskirts of a big city | -0.01 (0.00) | <0.0001 |
| A town or a small city | -0.01 (0.00) | <0.0001 |
| A country village | -0.01 (0.00) | <0.0001 |
| A farm or home in the countryside | -0.01 (0.00) | <0.0001 |
| *Financial vulnerability* |  |  |
| “Coping on present income” | -0.03 (0.00) | <0.0001 |
| "Finding it difficult on present income” | -0.03 (0.00) | <0.0001 |
| "Finding it very difficult on present income” | -0.04 (0.00) | <0.0001 |
| *Health vulnerability* |  |  |
| " Hampered in daily activities” | -0.02 (0.00) | <0.0001 |
| " A lot hampered in daily activities” | -0.01 (0.00) | <0.0001 |

*Note:* the above model was estimated using sampling weights and adjusted by country.
